# Recognizing a MIS-Chievous Cause of Acute Viral Gastroenteritis

**DOI:** 10.3389/fped.2021.748368

**Published:** 2021-10-29

**Authors:** Rohit Josyabhatla, Ankur A. Kamdar, Shabba A. Armbrister, Rhea Daniel, Konstantinos Boukas, Keely G. Smith, Melissa R. Van Arsdall, Kokila Kakarala, Anthony R. Flores, Audrey Wanger, Yuying Liu, Jon Marc Rhoads

**Affiliations:** ^1^Division of Gastroenterology, Department of Pediatrics, McGovern Medical School, University of Texas Health Science Center at Houston, Houston, TX, United States; ^2^Children's Memorial Hermann Hospital, Houston, TX, United States; ^3^Division of Rheumatology, Department of Pediatrics, McGovern Medical School, University of Texas Health Science Center at Houston, Houston, TX, United States; ^4^Division of Critical Care Medicine, Department of Pediatrics, McGovern Medical School, University of Texas Health Science Center at Houston, Houston, TX, United States; ^5^Division of Pediatric Hospital Medicine, Department of Pediatrics, McGovern Medical School, University of Texas Health Science Center at Houston, Houston, TX, United States; ^6^Division of Infectious Disease, Department of Pediatrics, McGovern Medical School, University of Texas Health Science Center at Houston, Houston, TX, United States; ^7^Department of Pathology and Laboratory Medicine, McGovern Medical School, University of Texas Health Science Center at Houston, Houston, TX, United States

**Keywords:** diarrhea, MIS-C multisystem inflammatory syndrome in children, zonulin, I-FABP2, claudin-3

## Abstract

Historically, children evaluated for vomiting and diarrhea secondary to viral enteritis have symptoms lasting 2–4 days and respond to supportive care, including oral rehydration and anti-emetics if required. Recently, within a 14-day timespan, we encountered three children with severe diarrhea who rapidly became dehydrated and went into hypotensive shock. Although SARS-CoV-2 molecular tests were negative by nasopharyngeal swab, all were later found to have MIS-C. This small case series underscores features reported in previous larger studies and emphasizes the rapid clinical evolution of this condition. We highlight the importance of early recognition of cardinal laboratory findings characteristic of MIS-C (i.e., lymphopenia, markedly elevated acute phase reactants, and hypoalbuminemia). We also show serologic evidence that the pathophysiological mechanism of SARS-CoV-2 related diarrhea may differ from other causes of dehydrating vomiting and diarrhea, with no serologic evidence of villus cell injury.

## Introduction

In recent decades, acute viral gastroenteritis has rarely progressed to hypotension in the United States (U.S.), although in developing countries, enterotoxin-secreting microbes and one particular virus, rotavirus, can induce a secretory diarrhea resulting in severe dehydration (generally with >10–20 stools/day) (1, 2). The variant of coronavirus disease 2019 (COVID-19) with multisystem inflammatory syndrome in children (MIS-C) has been reported to be associated with a variety of gastrointestinal symptoms—abdominal pain, diarrhea, vomiting, appendicitis, and bowel obstruction (3–5). We present a case series of three children who met the CDC criteria for MIS-C based on the presence of fever, multiorgan involvement, elevated laboratory markers (Table 1), lack of alternate etiology and presence of positive SARS-CoV-2 immunoglobulin G (IgG). The similarity in this cohort of children was the severe acute watery diarrhea leading to shock with rapid response to anti-inflammatory therapy, similar to another recent case series of children requiring inotropic support with acute watery diarrhea (6).

**Table 1:** Elevated levels of markers of inflammation in our cohort of patients.

**Laboratory test (with normal values)**	**Case 1**	**Case 2**	**Case 3**
Absolute lymphocyte count (cells/CMM) = 1,100–7,300	800	300	400
Albumin (g/dL) = 3.8–5.4	2.0	2.6	2.3
ESR (mm/h) = 0–10	32	40	50
CRP (mg/L) = <2.9	109	180	110
Procalcitonin (ng/mL) = 0.00–0.10	14.2	22.46	1.24
Lactic acid (mMol/L) = 0.5–2.2	3	2.5	1.5
Ferritin (ng/mL) = 5–204	N/A	583	760

*CDC definition of MIS-C (with the markers of inflammation that were elevated in our patients in bold) (5).*

- An individual aged <21 years presenting with fever, laboratory evidence of inflammation*, and evidence of clinically severe illness requiring hospitalization, with multisystem (>2) organ involvement (cardiac, renal, respiratory, hematologic, gastrointestinal, dermatologic, or neurological);

*AND*

*- No alternative plausible diagnoses*;

*AND*

*Positive for current or recent SARS-CoV-2 infection by RT-PCR, serology, or antigen test; or exposure to a suspected or confirmed COVID-19 case within the 4 weeks prior to the onset of symptoms*.

*Including, but not limited to, one or more of the following: **an elevated C-reactive protein (CRP), erythrocyte sedimentation rate (ESR)**, fibrinogen, **procalcitonin**, d-dimer, **ferritin, lactic acid** dehydrogenase (LDH), or interleukin 6 (IL-6), elevated neutrophils, **reduced lymphocytes, and low albumin**.

To better understand the pathophysiology of diarrhea we sequentially interrogated serologic markers of gut permeability (claudin-3 and zonulin), and villus integrity (I-FABP2). Zonulin is a regulator of intestinal epithelial tight junctions (TJ) (7, 8) and claudin-3 serves as a measure of TJ integrity (9, 10). I-FABP has been used to assess villus injury (11, 12). All three markers have been reported to be elevated when enterocytes are damaged by inflammation, ischemia, or infection (8–12).

## Methods

### Case Studies and Ethical Approval

We received approval from our Institutional Review Board to report these cases and to perform analysis of blood markers of intestinal damage (HSC-MS-21-0204).

#### Case 1

An 11-year old Asian male, presented in hypovolemic shock with 4 days of non-bloody, watery diarrhea with mucus (up to 10 bowel movements/day), associated with diffuse abdominal pain and nausea. He had subjective fever and shortness of breath for 1 day. He was hypotensive and tachycardic on exam and was admitted to the intensive care unit (ICU), where he required aggressive fluid resuscitation and pressor support with epinephrine and norepinephrine.

His SARS-CoV-2 exposure was unclear. He tested negative for SARS-CoV-2 by molecular assay. He did not have classic signs of Kawasaki Disease including rash, conjunctivitis, erythematous cracked lips, adenopathy, or swelling of his hands or feet. Laboratory studies showed lymphopenia (absolute lymphocyte count [ALC] of 800/μL) and hypoalbuminemia (2.0 g/dL) with elevated inflammatory markers: procalcitonin (14.2 ng/mL), lactic acid (LA) (3 mMol/L), erythrocyte sedimentation rate (ESR) (32 mm/h), and C-reactive protein (CRP) (109 mg/L). Renal function was normal. Although he had mild elevation of transaminases—alanine aminotransferase (ALT) 106 unit/L, aspartate aminotransferase (AST) 201 unit/L; prothrombin time was normal, and viral hepatitis screen was negative. Fecal occult blood was positive. His blood and urine cultures, respiratory viral pathogen panel (RPP), stool gastrointestinal pathogen panel, and *C. difficile* polymerase chain reaction (PCR) assay were negative. Troponin-I (1.25 ng/mL), pro-brain natriuretic peptide (Pro-BNP) (6,865 pg/mL), and creatine kinase (CK) (549 units/L) were all elevated. Blood samples obtained on admission were retrospectively found to be positive for SARS-CoV-2 immunoglobulin G (IgG).

**Figure 1:**
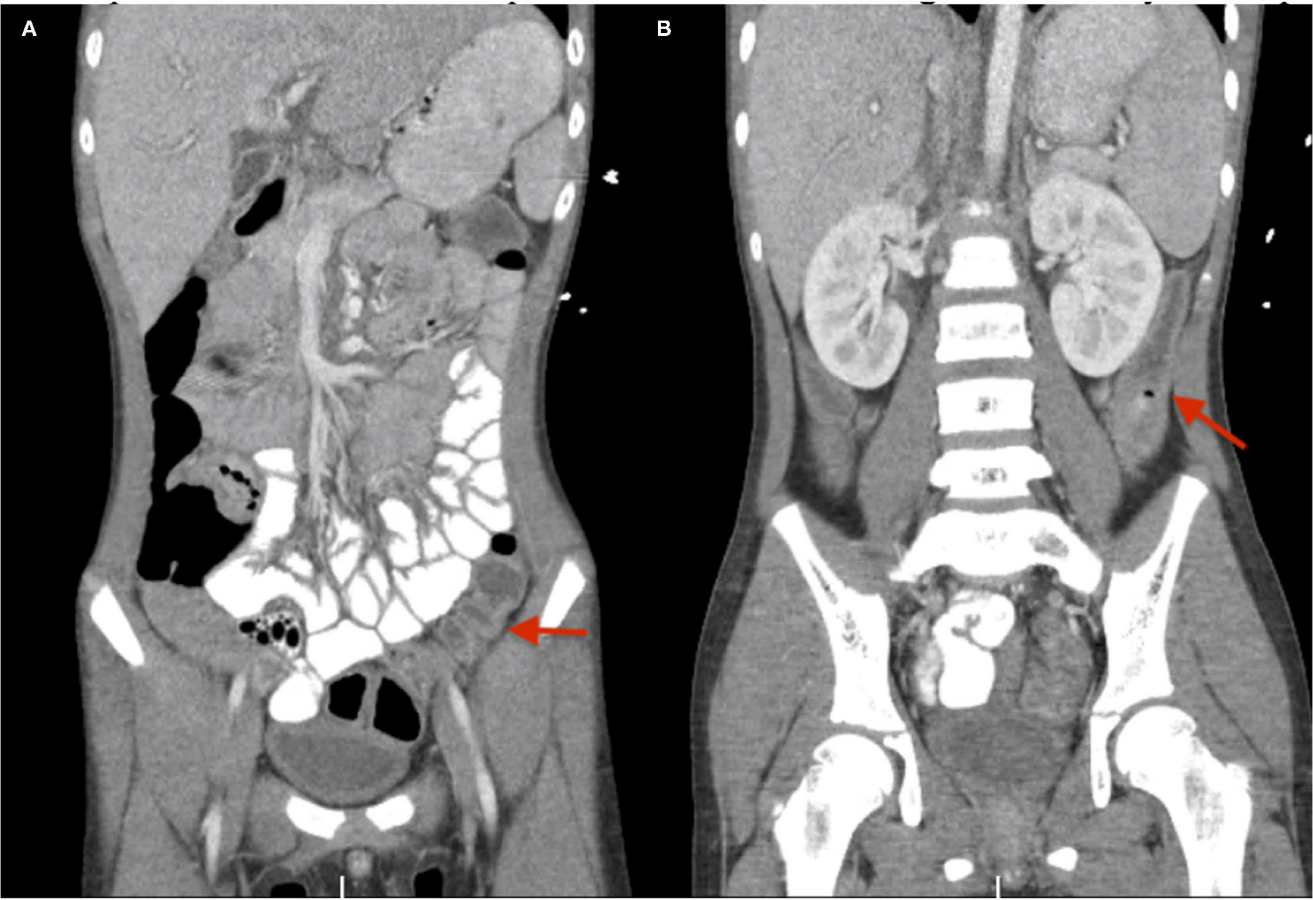
**(A,B)** CT abdomen pelvis with contrast for case #1. Red arrows depict the descending colon edema.

Echocardiogram showed mild right coronary artery dilation with no ventricular dysfunction. Computed tomography (CT) of the abdomen and pelvis with contrast showed moderate gallbladder wall edema with pericholecystic fluid, mild periportal edema, urinary bladder wall thickening, and descending colon edema (Figure 1).

In addition to crystalloid fluids and vasopressors, he was given albumin replacement, metronidazole and ceftriaxone. He was diagnosed with MIS-C and treated with intravenous immunoglobulin (IVIG), high dose intravenous (IV) methylprednisolone (30 mg/kg daily for 5 days) and anakinra, an interleukin (IL)-1 receptor antagonist (6 mg/kg/day IV for 5 days). He was anticoagulated with enoxaparin [1 mg/kg subcutaneous (SC) injection daily] during hospitalization. He had dramatic improvement and was discharged after 10 days of hospitalization on aspirin (81 mg oral daily).

#### Case 2

A 6-year-old girl presented with a 5-day history of daily fevers and chills up to 101°F and 3-days of diffuse abdominal pain and vomiting. Her symptoms started after attending a party. She was not able to retain any food, prompting her visit to the emergency department (ED). She denied diarrhea or respiratory symptoms. There were multiple COVID-19 cases at her school. Exam was remarkable for abdominal tenderness with rebound tenderness and chapped lips.

**Figure 2:**
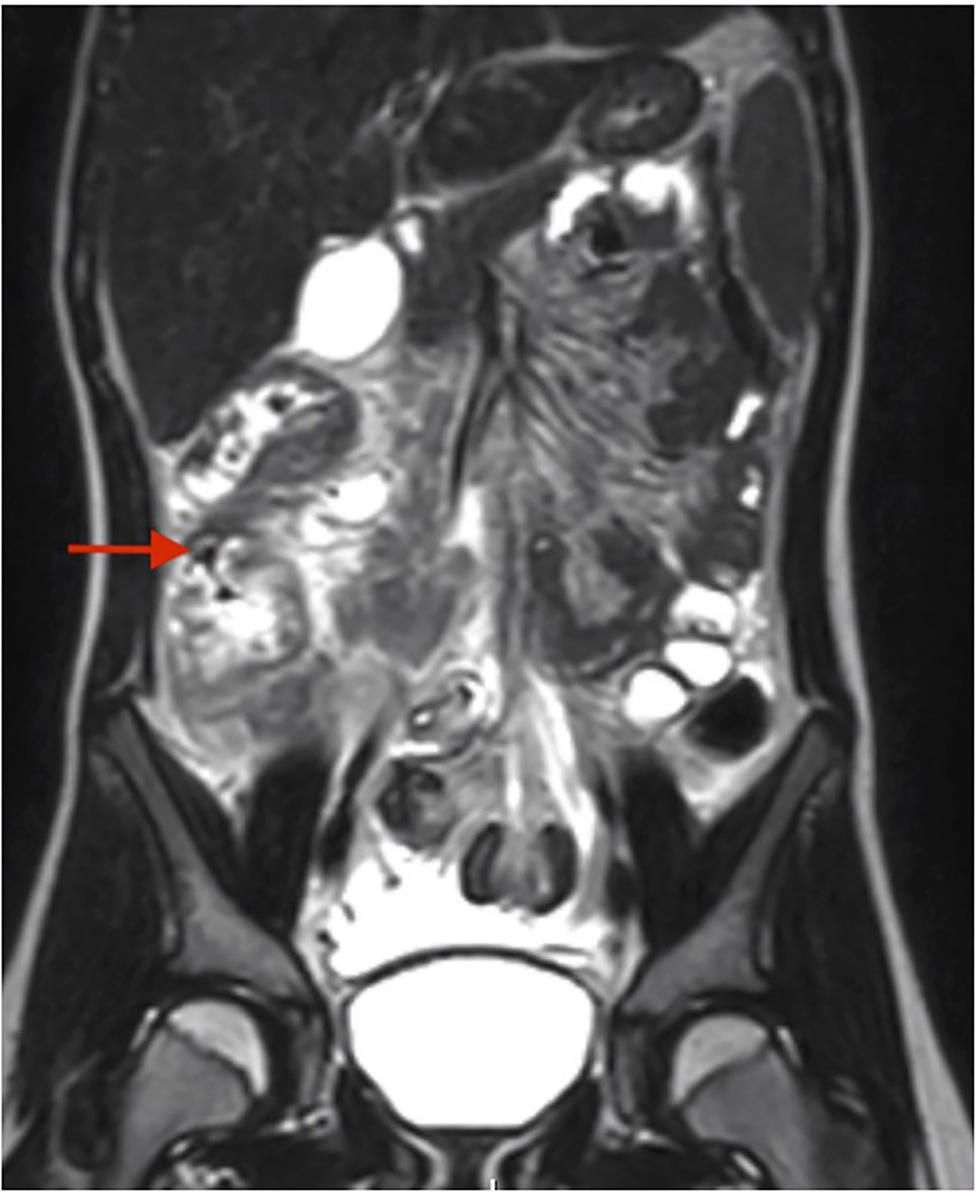
MR enterography for case #2. Red arrow depicts the mural edema and circumferential thickening of the cecum and ascending colon.

A right lower quadrant ultrasound showed diffusely increased fat echogenicity, decreased bowel peristalsis, mesenteric lymphadenopathy, and free fluid in the pelvis, concerning for appendicitis. Magnetic resonance imaging showed a fluid-filled appendix measuring 0.6 cm and mural edema and circumferential thickening involving the cecum, proximal ascending colon and distal terminal ileum, with fat stranding and moderate amount of free fluid (Figure 2). She was admitted to the hospital for appendectomy and started on pipercillin-tazobactam. Operative findings revealed a grossly normal-appearing appendix with free fluid in the pelvis. The ileocecal fold was inflamed and adherent to the terminal ileum. The pathology report, however, showed acute purulent appendicitis.

Post-operatively the patient became febrile, with vomiting and new-onset severe watery diarrhea, resulting in shock, ICU admission, and aggressive fluid resuscitation. Chest x-ray showed mild diffuse hazy opacities in bilateral lung fields. Echocardiogram showed mild right coronary artery dilation.

Labs revealed normal electrolytes and renal function, a low albumin of 2.6 g/dL, lymphopenia [ALC 300/μL with white blood cell count (WBC) 10,800/μL], and elevated CRP (180 mg/L), ESR (40 mm/h), LA (2.5 mMol/L), procalcitonin (22.46 ng/mL), ferritin 583 (ng/mL), and pro-BNP (12,800 pg/mL). SARS-CoV-2 molecular test was negative, but serum SARS-CoV-2 IgG was positive. Stool tests revealed a negative *C. difficile* PCR and negative fecal occult blood, with no leukocytes present. Blood, urine, and throat cultures were negative. Fecal gastrointestinal pathogen profile was negative. Given the combination of fever, multisystem involvement, elevated inflammatory markers, and positive SARS-CoV-2 IgG, the patient was diagnosed with MIS-C and was treated with high dose methylprednisolone (30 mg/kg/day IV for 5 days) and anakinra (9 mg/kg/day IV for 5 days). She was anticoagulated with enoxaparin (1 mg/kg SC injection daily) during hospitalization, quickly responded to treatment, and was discharged home on aspirin (81 mg oral daily).

#### Case 3

A 9-year-old boy with a history of asthma was admitted with fever, abdominal pain, vomiting and watery diarrhea for 4 days. Due to an unprecedented snowstorm in Houston, TX, prohibiting safe travel and in-person evaluation, he was prescribed azithromycin, dicyclomine, and loperamide by his primary care provider. He remained febrile with poor oral intake and developed reduced urine output the next day, prompting ED evaluation. He reported using his nebulizer for shortness of breath over the preceding 4 days. He and family members had been SARS-CoV-2 positive 4 weeks prior to his ED presentation but had all fully recovered. SARS-CoV-2 molecular testing on admission was negative.

On exam, he was dehydrated with tachycardia and a wide pulse pressure. He was given two normal saline fluid boluses. Physical exam did not reveal a rash or mucositis. Initial labs showed normal renal function, an albumin of 3.3 g/dL (repeat 2.3 g/dL), lactate dehydrogenase (LDH) 299 units/L, lipase 46 units/L, lactic acid 1.5 mmol/L, procalcitonin 1.24 ng/mL, and ferritin 760 ng/mL. WBC was 4.5 K/cmm with lymphopenia (ALC 400/μL). ESR (50 mm/h), CRP (110 mg/L), INR (1.07), and pro-BNP (1,830 ng/mL) were elevated. Troponin-I was normal. Urinalysis showed a specific gravity of 1.044, with trace ketonuria, pyuria, and proteinuria. Although fecal SARS-CoV-2 antigen test was negative, serum SARS-CoV-2 IgG antibody was positive.

An abdominal ultrasound showed a small amount of ascites; the gallbladder wall was mildly thickened. Echocardiogram showed trace tricuspid regurgitation without coronary aneurysms or cardiac dysfunction. After blood and urine cultures were obtained, he was empirically started on intravenous broad-spectrum antibiotics. He was initially admitted to the acute medical floor but rapidly decompensated, developing hypotension and respiratory distress that required ICU admission. He was fluid resuscitated and required positive pressure ventilation. He was diagnosed with MIS-C and started on intravenous methylprednisolone (30 mg/kg/dose for 5 days) and anakinra (10 mg/kg/day IV for 5 days). He had rapid improvement in symptoms and was discharged 5 days later. He was anticoagulated with enoxaparin (1 mg/kg SC injection daily) during hospitalization.

### Enzyme-Linked Immunosorbent Assay (for Plasma Levels of Claudin-3, Intestinal Fatty Acid Binding Protein, and Zonulin

Human Claudin-3 ELISA kit was purchased from Novus Biologicals, Human I-FABP Quantikine ELISA kit from R&D Systems, and Human Zonulin ELISA kit from Biomatik Corporation. The assays were performed according to the manufacturers' protocol. The detection ranges were 0.31–20 ng/mL for claudin-3, 2.12–6.21 pg/mL for I-FABP and 0.625–40 ng/mL for zonulin. Samples required dilutions as necessary, and the results were multiplied by dilution factors.

## Results

### Serum Levels Evaluating Gut Integrity

We obtained 5–7 sequential sera from each child with MIS-C and determined levels of three markers of intestinal integrity. These markers were compared to plasma levels from age-matched healthy controls (Table 2).

**Table 2:** Study population demographics.

	**Cases (***n*** = 3)**	**Controls (***n*** = 17)**
Age (mean with SD)	8.67 (2.516)	11.12 (2.147)
Male (%)	66.67	45.06
**Race (%)**		
Caucasian	33.33	82.35
African American	33.33	11.76
Asian	33.33	5.88
**Ethnicity**		
Hispanic	33.33	35.29

**Figure 3:**
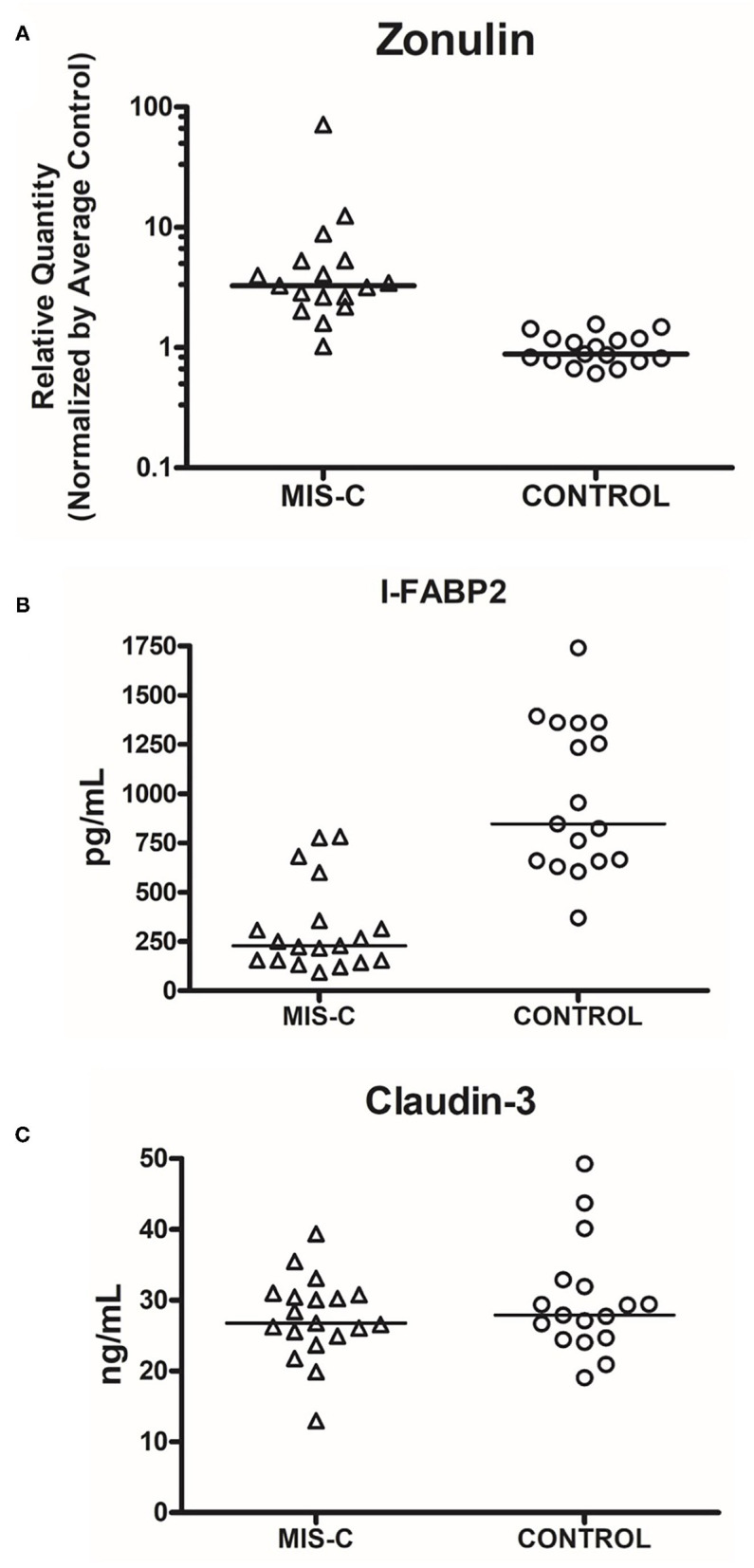
Comparison of serum levels of markers of gut injury in cases vs. controls. **(A)** Zonulin (*P* < 0.0001, Cases vs. Controls, Mann–Whitney Test). **(B)** I-FABP2 (*P* < 0.0001, Cases vs. Controls, Mann–Whitney Test); **(C)** Claudin-3 (*P* = 0.6573, Cases vs. Controls, Mann–Whitney Test). The line represents median. Each dot represents each sample that was measured.

**Figure 4:**
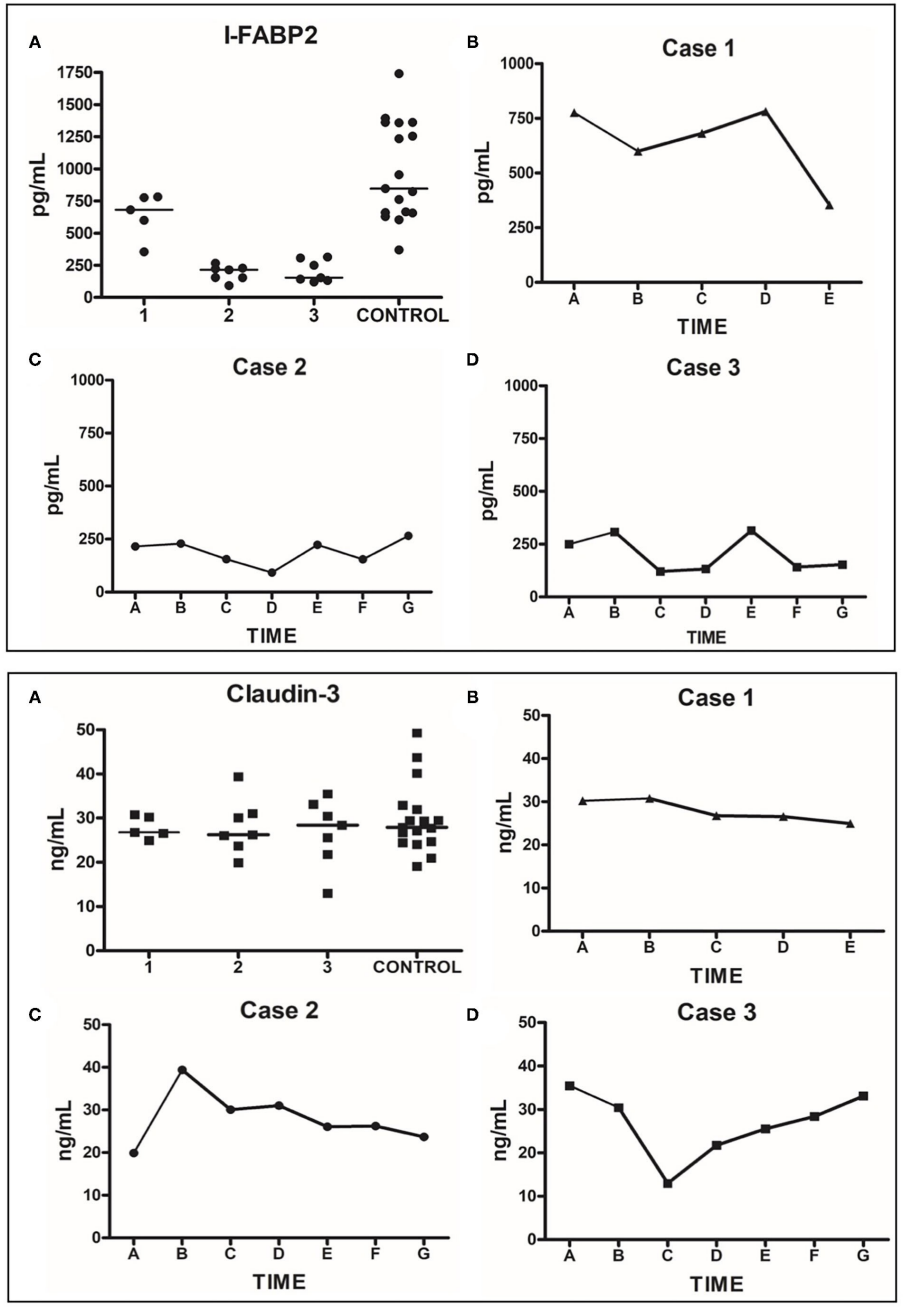
Human plasma I-FABP2 and Claudin-3 analysis. Top Panel: I-FABP2. **(A)** Grouped values for each patient during their hospitalization compared to grouped values of controls. **(B)** Daily sequential values for Case 1; time point A represents day 7 of hospitalization (day 3 of anakinra), when patient was clinically stable with resolution of diarrhea; the subsequent points are from daily samples obtained over the next 5 days until the day of discharge. **(C)** Daily sequential values for Case 2; time point A represents day 2 of hospitalization (day prior to onset of diarrhea, day after the appendectomy); the subsequent points are from daily samples obtained over the next 7 days when the diarrhea developed— requiring treatment with anakinra and steroids leading to resolution of symptoms; the last sample was from the day of discharge. **(D)** Daily sequential values for Case 3; time point A represents the day 2 of hospitalization when the patient was diagnosed with MIS-C and received the first dose of anakinra and steroids. The subsequent points are from daily samples obtained over the next week of his hospitalization, with the last sample obtained on the day of discharge. Bottom Panel: Claudin-3. **(A–D)** described the same as Top Panel **(A–D)**, respectively.

Zonulin levels were found to be significantly elevated in these patients compared to controls (Figure 3, *P* < 0.0001). I-FABP levels were found to be significantly lower than controls (Figure 3, *P* < 0.0001). There were no significant differences in the level of claudin-3 between patients and controls (Figure 3, *P* = 0.6573). Sequential daily analysis over a 5–7-day period during hospitalization did not reveal a clear trend in the levels of I-FABP or claudin-3, despite obvious symptomatic improvement and resolution of diarrhea (Figure 4).

## Discussion

Gastroenterologists are often consulted to aid in the diagnosis and management of infectious causes of diarrhea. Generally, enterotoxigenic bacteria cause the most severe fluid loss and dehydration. For example, with cholera, severe diarrhea and vomiting can lead to circulatory collapse (13); however, cholera is rare in the U.S., and the children in this series did not consume raw seafood. In the three cases of MIS-C presented, the diarrhea resolved within 1–2 days after initiation of anti-inflammatory therapy. In all three patients although the nasopharyngeal swab was negative for SARS-CoV-2, the patients described were positive by serology (SARS-CoV-2 IgG-positive). This is in addition to the fever and multi-organ involvement is consistent with MIS-C as per the CDC definition (5).

This small case series, encountered about a year after the SARS-CoV-2 outbreak began, serves as a reminder to be aware of diarrheal disease secondary to MIS-C, in patients with negative SARS-CoV-2 antigen tests. Only one of the three children presented had mucositis, with severely fissured and cracking chapped lips. Another disorder that can lead to shock is hemolytic uremic syndrome, which is often caused by *E. coli* O157:H7; however, this condition leads to a classic triad of thrombocytopenia, microangiopathic hemolytic anemia, and acute kidney injury (14) which was not seen in any of our cases. Rotavirus infection is known to lead to dehydration in up to 10% of infants. However, this condition is most severe in young infants and toddlers, may cause severe and prolonged metabolic acidosis (15) and has been much less prevalent in the decade following routine rotavirus vaccination.

The data are limited regarding optimal treatment for MIS-C although in a recent systematic review, IVIG and steroids were used in 76 and 57% of all patients, respectively, while anakinra was used in 8% (16). From a therapeutic perspective, one child in this report received IVIG, and all three were treated with methylprednisolone and the interleukin-1 inhibitor anakinra. No patients developed coronary aneurysms or cardiac dysfunction on echocardiogram evaluation. None required mechanical ventilation or extracorporeal membrane oxygenation (ECMO). Given the clinical similarities of MIS-C and Kawasaki Disease (KD), the general treatment approach for MIS-C has been to follow the treatment approach for KD, which is IVIG, and in severe cases, steroids and cytokine inhibition. Noteworthy, patients with MIS-C tend to develop shock and cardiac abnormalities more frequently than those with KD (16).

Our report is much smaller than several comprehensive pediatric reviews of MIS-C. A report from Spain compared MIS-C with non-MIS-C COVID-19 patients and found in those with MIS-C more respiratory distress, fever, diarrhea, vomiting, abdominal pain, and shock, with requirement for inotropic support (17). Another Spanish group reported KD-like features in about half of MIS-C cases (18). A multicenter review of MIS-C (*n* = 78 cases) conducted in the United Kingdom emphasized fever in 100%, shock in 87%, abdominal pain in 62%, and vomiting/ diarrhea in 64% of patients. In that series, three needed ECMO, two died, and coronary aneurysms were discovered in 36% of the patients (19). Two reports came from the New York City outbreak. In one, the majority had G.I. symptoms, which in one case led to ileal obstruction mandating ileocolonic resection with ileostomy; the resected tissue showed severe transmural inflammation and venous microthrombi. Only 1 of 35 had a KD-like presentation of MIS-C, and the survival rate with aggressive treatment was 100% (20). Another New York report of 44 children and young adults documented diarrhea in 40%, abdominal pain in 75%, vomiting in 57%, rash in 71%, and conjunctivitis in 32%. Again, mortality was nil, although one child required renal transplantation (21). Systematic reviews have confirmed the same cluster of gastrointestinal symptoms, now in series of hundreds of children (3, 4). Most recently, Sahn et al. (20) reported that in a series of 35 children with this condition, abdominal pain, and diarrhea occurred in 97% of patients in their series. In one child who went to surgery, histological evaluation showed that the inflammation appeared to originate in the subendothelial space, with venous microthrombi and transmural lymphocytic infiltration and necrotizing lymphadenitis.

We attempted to better understand the pathophysiology of the disease process using three different intestinal markers as described above. Zonulin is a modulator of intestinal epithelial tight junctions (7). Typically, triggered by intestinal bacteria and gliadin, it is released from enterocytes and is believed to cause breakdown of zonula occludens (ZO-1) leading to a disruption of the intestinal tight junctions (TJ). Zonulin has been studied in several intestinal disorders, including celiac disease, diarrhea-predominant irritable bowel syndrome, inflammatory bowel disease, non-celiac gluten sensitivity, and environmental enteric dysfunction. Plasma levels of zonulin were elevated in all these conditions compared to healthy individuals (8). In a recent study by Yonker et al. (22), zonulin levels in patients with MIS-C were found to be significantly higher than in children with COVID-19 and healthy controls. The investigators found that elevated zonulin levels correlated with spike protein antigenemia. Furthermore, they found that the use of larazotide acetate, a zonulin antagonist, in one patient resulted in improvement in antigen levels and clinical symptoms. Our cases were similarly found to have significantly elevated plasma zonulin levels compared to controls.

Claudin-3 has a sealing function at the intestinal epithelial TJ (23). It has been shown to be involved in maintaining the TJ barrier properties of the intestinal epithelial cells (9, 10). Plasma claudin-3 levels were noted to be increased significantly in pediatric congenital heart disease patients following cardiopulmonary bypass (12, 24). In addition, urinary claudin-3 levels were significantly higher in neonates with necrotizing enterocolitis than in neonates with other diagnoses (25). We did not find a difference in the levels of claudin-3 in our patients with MIS-C compared to healthy controls.

I-FABP serves as a sensitive marker of enterocyte injury. Plasma I-FABP levels rise in conditions such as untreated celiac disease, shock, and abdominal trauma (26–28). In our patients with MIS-C, we hypothesized that the levels of I-FABP would be elevated, because each child developed hypovolemic shock. To our surprise, the I-FABP levels were significantly lower in the cases as compared to healthy controls. These findings are in line with a study conducted by Guedj et al. (29), where the investigators compared plasma I-FABP levels in COVID-19 patients with pneumonia to patients who had pulmonary disease but not COVID-19 and to patients with abdominal pain but not COVID-19. The level of I-FABP was significantly lower in patients with COVID-19 compared to patients with pulmonary disease and abdominal pain who did not have COVID-19. All COVID-19 patients had pneumonia (*n* = 28), and 11 of them also had diarrhea. The lower than normal I-FABP levels in two of our cases (both consistently <100 pg/mL) could be due to blunted (shortened) villi without inflammation, as seen in porcine coronavirus transmissible gastroenteritis (TGE) (30). In the TGE piglet coronavirus model, gut permeability to macromolecules was increased only briefly—for 12 h—following infection and rapidly returned to normal the second day.

## Conclusions

Based on our findings of normal claudin 3 and I-FABP, and elevated zonulin in this small case series of diarrhea predominant MIS-C, we believe that the diarrhea is due to a leaky gut triggered by increased zonulin. The trigger for increased zonulin levels could be inflammation localized to the gut endothelium, microvascular inflammation, and/or a dysbiotic microbiome in patients after recovery from acute COVID-19. Although zonulin causes disassembly of the TJ, claudin-3 does not appear to be its target. Additionally, there does not appear to be ongoing villus injury in these patients.

MIS-C may therefore represent an “immunological storm” of the gastrointestinal tract, with fluid and electrolyte hypersecretion, leading to shock, which resolves with anti-inflammatory treatment. Note that in a previous study of piglet coronavirus enteritis, corticosteroid treatment unexpectedly resulted in accelerated recovery of mucosal damage (31). Intestinal biopsy evidence in support of this concept would be helpful, but the rapid response to immune suppression strongly supports this concept and appears essential to optimal care.

## Data Availability Statement

The original contributions presented in the study are included in the article/supplementary material, further inquiries can be directed to the corresponding author/s.

## Ethics Statement

The studies involving human participants were reviewed and approved by Committee for the Protection of Human Subjects, The University of Texas Health Science Center at Houston. Written informed consent from the participants' legal guardian/next of kin was not required to participate in this study in accordance with the national legislation and the institutional requirements.

## Author Contributions

RJ, SA, YL, and JR participated in the study design, patient care, data collection, analysis of samples, writing, and reviewing the manuscript. RD, AK, KB, KS, MV, KK, AF, and AW participated in patient care, writing, and reviewing the manuscript. All authors contributed to the article and approved the submitted version.

## Funding

This study was provided by the Department of Pediatrics, Division of Pediatric Gastroenterology and the Children's Memorial Hermann Hospital.

## Conflict of Interest

The authors declare that the research was conducted in the absence of any commercial or financial relationships that could be construed as a potential conflict of interest.

## Publisher's Note

All claims expressed in this article are solely those of the authors and do not necessarily represent those of their affiliated organizations, or those of the publisher, the editors and the reviewers. Any product that may be evaluated in this article, or claim that may be made by its manufacturer, is not guaranteed or endorsed by the publisher.
